# ATP as a multi-target danger signal in the brain

**DOI:** 10.3389/fnins.2015.00148

**Published:** 2015-04-28

**Authors:** Ricardo J. Rodrigues, Angelo R. Tomé, Rodrigo A. Cunha

**Affiliations:** ^1^CNC - Center for Neuroscience and Cell Biology, University of CoimbraCoimbra, Portugal; ^2^Institute for Interdisciplinary Research, University of CoimbraCoimbra, Portugal; ^3^Department of Life Sciences, Faculty of Sciences and Technology, University of CoimbraCoimbra, Portugal; ^4^Faculty of Medicine, University of CoimbraCoimbra, Portugal

**Keywords:** ATP, adenosine, P2 receptors, P1 receptors, ecto-nucleotidases, P2X7 receptor, P2Y1 receptor, A_2A_ receptor

## Abstract

ATP is released in an activity-dependent manner from different cell types in the brain, fulfilling different roles as a neurotransmitter, neuromodulator, in astrocyte-to-neuron communication, propagating astrocytic responses and formatting microglia responses. This involves the activation of different ATP P2 receptors (P2R) as well as adenosine receptors upon extracellular ATP catabolism by ecto-nucleotidases. Notably, brain noxious stimuli trigger a sustained increase of extracellular ATP, which plays a key role as danger signal in the brain. This involves a combined action of extracellular ATP in different cell types, namely increasing the susceptibility of neurons to damage, promoting astrogliosis and recruiting and formatting microglia to mount neuroinflammatory responses. Such actions involve the activation of different receptors, as heralded by neuroprotective effects resulting from blockade mainly of P2X7R, P2Y1R and adenosine A_2A_ receptors (A_2A_R), which hierarchy, cooperation and/or redundancy is still not resolved. These pleiotropic functions of ATP as a danger signal in brain damage prompt a therapeutic interest to multi-target different purinergic receptors to provide maximal opportunities for neuroprotection.

## Introduction

Intracellular adenosine 5′-triphosphate (ATP) plays several pivotal roles, namely in energy transfer (Lipmann, 1941). Hence, the proposal by Burnstock (1972) that ATP was released to function as an extracellular signal was controversial. However, this concept is supported by the identification of mechanisms of ATP release, of ecto-enzymes metabolizing ATP named ecto-nucleotidases, and of purinergic receptors. ATP can trigger biological effects *per se* through the activation of P2 receptors (P2R) or through its ecto-nucleotidase metabolites ADP activating some P2R and adenosine through P1R activation (Ralevic and Burnstock, 1998). Cloning identified seven P2XR subunits P2X1-7, forming functional homomeric or heteromeric ionotropic receptors activated by ATP (Khakh and North, 2012) and eight different metabotropic P2YR (P2Y1,2,4,6,11,12,13,14) exhibiting a different sensitivity to ATP (P2Y11), ADP (P2Y1,12,13), UTP/ATP (P2Y2,4), UDP (P2Y6), or UDP-glucose (P2Y14) (Abbracchio et al., 2006), whereas adenosine P1R family comprises A_1_, A_2A_, A_2B_, and A_3_ metabotropic receptors, identified by convergent molecular, biochemical and pharmacological data (Fredholm et al., 2011).

ATP is stored in synaptic and in astrocyte vesicles, but it can be released from different cell types, namely nerve terminals, dendrites, and axons from neurons (Pankratov et al., 2006; Fields, 2011), astrocytes (Koizumi, 2010) and microglia (Imura et al., 2013; George et al., 2015) through multiple pathways (Bodin and Burnstock, 2001). Also, purinergic receptors display a widespread brain expression both in neuronal or non-neuronal cells such as astrocytes, microglia or endothelial cells (Fredholm et al., 2005; Fields and Burnstock, 2006). Accordingly, multiple roles have been attributed to extracellular ATP. ATP can act as a neurotransmitter, since P2XR-mediated ATPergic transmission has been found in central synapses (Edwards et al., 1992; Bardoni et al., 1997; Nieber et al., 1997; Pankratov et al., 1998, 2002; Mori et al., 2001). ATP is also a controller of inflammation (Idzko et al., 2014), with multiple actions on microglia (Koizumi et al., 2013) and its consequences on astrocytes and neurons. ATP and adenosine both regulate oligodendrocyte differentiation and myelination (Agresti et al., 2005; Rivkees and Wendler, 2011) in an activity-dependent manner (Fields, 2006). Moreover, purines modulate astrocytic function and sustain Ca^2+^-waves, the substrate of glial excitability and intercellular communication (Guthrie et al., 1999; Koizumi, 2010) to influence synaptic activity (Zhang et al., 2003; Jourdain et al., 2007; Franke et al., 2012). In fact, it is mostly concluded that ATP acts as a synaptic neuromodulator through presynaptic regulation of neurotransmitter release, by postsynaptic regulation of other receptors or of intrinsic neuronal excitability, with an impact in synaptic plasticity (Cunha and Ribeiro, 2000; Khakh, 2001; Halassa et al., 2009).

The variety of purinergic receptors and their widespread region- and cell-specific expression pattern and actions places purinergic signaling as a major system for integration of functional activity between neurons, glial and vascular cells in the brain as heralded by the role of purines (ATP and adenosine) in neuron-neuron, astrocyte-neuron, oligodendrocyte-neuron and/or microglia/neuron bi-directional communication (Fields and Burnstock, 2006; Butt, 2011). Moreover, the different sensitivities of the different receptors to their different ligands (ATP, ADP, adenosine) displaying spatial and temporal fine-tuned gradients (Zhang et al., 2003; Cunha, 2008), endows purinergic signaling with unique features adapted to control brain networks. Not surprisingly, the dysfunction of this purinergic system is closely associated with brain disorders and we will now exploit the concept that ATP acts as a danger signal, implying an abnormal and sustained elevation of extracellular ATP levels in brain dysfunction and the involvement of purine receptors, namely P2X7R (ATP), P2Y1R (ADP) and A_2A_R (adenosine), in brain damage.

## Sustained increase of extracellular ATP levels in brain pathology

**Figure 1:**
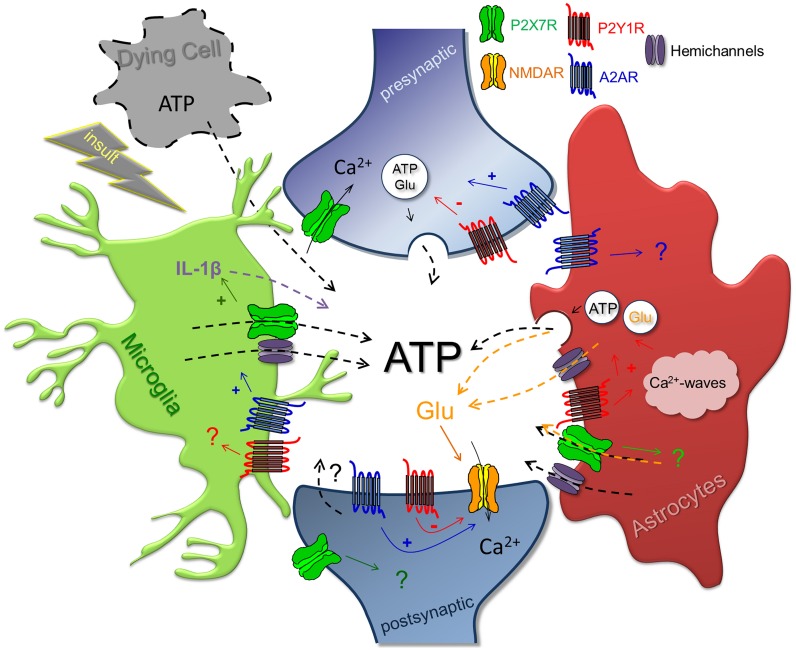
**Integrated view of the purinergic signaling in brain disorders**. In addition to the leakage of ATP through damaged cell membrane from injured or dying cells, evolution has assured multiple mechanisms from different sources to place ATP in the extracellular milieu as a danger signal in the brain. Interestingly, this increase is self-sustained: activation of P2X7R induces the release of ATP either directly through its channel or by exocytotic or non-exocytotic mechanisms (e.g., hemichannels); P2Y1R induces the release of ATP from astrocytes; A_2A_R controls the release of ATP from microglia and presynaptic terminals. Once in the extracellular millieu, ATP seems to contribute to neurotoxicity through an integrated action through P2X7R, P2Y1R, and A_2A_R. P2X7R: it is well-established that P2X7R antagonism is beneficial by preventing the neurotoxic processing and release of IL-1β from microglia; yet a deleterious action through astrocytes namely through the regulation of glutamate levels or pro-inflammatory cytokines, or a direct neurotoxic action cannot be discarded. P2Y1R: the contribution of P2Y1R to brain demises has been mainly associated to astrocytic reactivity through Ca^2+^-waves and through an astrocytic-driven release of glutamate; this may be further promoted by direct actions on neuronal and synaptic function. A_2A_R: there is gain of function of A_2A_R particularly targeted to synapses in different brain disorders, where A_2A_R either with a presynaptic or postsynaptic locus of action, has been associated to synaptic dysfunction/loss; the precise mechanisms remain to be identified.

There is growing evidence for a rapid increase of the extracellular ATP levels upon noxious brain conditions such as trauma (Wang et al., 2004; Davalos et al., 2005; Franke et al., 2006; Choo et al., 2013), hypoxia/ischemia (Lutz and Kabler, 1997; Jurányi et al., 1999; Melani et al., 2005) or epilepsy-associated seizures (Wieraszko et al., 1989; see Dale and Frenguelli, 2009). The sustained nature of the enhanced extracellular levels of purines (ATP and adenosine) in brain dysfunction is indicative of regulated mechanisms of ATP release rather than simple ATP leakage. However, neither the cellular source nor the mechanism of ATP release upon noxious brain conditions has yet been clarified. Neurons can release ATP either through a vesicular release (White, 1977; Pankratov et al., 2006) mostly occurring at high frequency of firing (Wieraszko et al., 1989; Cunha et al., 1996a) or upon anoxic or spreading depolarization (Frenguelli et al., 2007). Astrocytes (Florian et al., 2011; Bennett et al., 2012) and microglia (Kim et al., 2007; Sanz et al., 2009) can also release purines upon brain dysfunction through vesicular release (Coco et al., 2003; Bowser and Khakh, 2007; Imura et al., 2013) and/or other mechanisms namely pannexin and/or connexin channels (Bao et al., 2004; Reigada et al., 2008; Iwabuchi and Kawahara, 2011), which have been proposed as a target for neuroprotection (Shestopalov and Slepak, 2014). In other cells, ATP release through lysosomal-dependent vesicles (Zhang et al., 2007) and/or from pannexin channels (Bennett et al., 2012) from autophagic (Wang et al., 2013) or apoptotic cells (Sandilos et al., 2012; Xiao et al., 2012) acts as a find-me signal (Elliott et al., 2009) (Figure 1).

## Purinergic receptors in brain pathology

The concept of ATP as a danger signal implies the release of ATP but also the involvement of purinergic receptors in brain disorders, which has mostly been documented for P2X7R, P2Y1R, and A_2A_R.

### P2X7 receptor

P2X7R have a lower affinity for ATP (0.1–1 mM) compared to other P2XR (*EC*_50_ = 1–10 μ M) (Surprenant and North, 2009), suggesting that their activation mostly occurs in pathological conditions associated to enhanced extracellular ATP levels. This is supported by the well-documented increase of P2X7R levels and P2X7R gain-of-function to control different brain disorders, from trauma or metabolic stress (Cavaliere et al., 2004; Franke et al., 2004; Melani et al., 2006; Arbeloa et al., 2012; Kimbler et al., 2012) to Alzheimer's disease (AD; Parvathenani et al., 2003; McLarnon et al., 2006; Díaz-Hernández et al., 2012; Murphy et al., 2012), Parkinson's disease (PD; Marcellino et al., 2010; Carmo et al., 2014a), Huntington's disease (HD; Díaz-Hernández et al., 2009) epilepsy (Solle et al., 2001; Vianna et al., 2002; Rappold et al., 2006; Avignone et al., 2008; Doná et al., 2009; Engel et al., 2012; Jimenez-Pacheco et al., 2013), prion disease (Takenouchi et al., 2007), and multiple sclerosis (MS, Matute et al., 2007; Sharp et al., 2008; Grygorowicz et al., 2011). Increased P2X7R levels have been also reported in human brain tissue of patients with temporal lobe epilepsy (Fernandes et al., 2009; Padrão et al., 2011), MS or AD (Narcisse et al., 2005; McLarnon et al., 2006; Yiangou et al., 2006).

P2X7R up-regulation has been mainly associated with microgliosis, since P2X7R promote neuronal death through microglia-derived interleukin-1β (IL-1β) (Ferrari et al., 1996; Chakfe et al., 2002; Skaper et al., 2006; Bernardino et al., 2008; Takenouchi et al., 2009) or production of reactive oxygen species (Parvathenani et al., 2003; Skaper et al., 2006; Lee et al., 2011). In AD, P2X7R are predominantly up-regulated in microglia around β-amyloid (Aβ) plaques in mice (Parvathenani et al., 2003; Lee et al., 2011) and humans (McLarnon et al., 2006) and Aβ triggers IL-1β secretion from microglia in a P2X7R-dependent manner (Sanz et al., 2009). A similar gain of function of P2X7R in formatting microglia responsiveness has been observed after ischemia (Franke et al., 2004), MS (Yiangou et al., 2006), prion disease (Takenouchi et al., 2007), PD (Marcellino et al., 2010) or upon *status epilepticus* (Rappold et al., 2006; Avignone et al., 2008; Kim et al., 2009; Choi et al., 2012; Engel et al., 2012), where P2X7R blockade/deletion reduces seizure severity during *status epilepticus* (Solle et al., 2001; Engel et al., 2012; Jimenez-Pacheco et al., 2013). P2X7R have also been linked to psychiatric disorders, as heralded by the association of P2X7R polymorphisms with major depression (Lucae et al., 2006; Hejjas et al., 2009) and by the anti-depressive behavior of P2X7R KO mice (Basso et al., 2009; Csölle et al., 2013), in line with the ability of IL-1β to induce depression-like behavioral changes (Pollak and Yirmiya, 2002; Anisman et al., 2005).

Besides this major role on overactivation of microglia, P2X7R are also up-regulated in reactive astrocytes and in neurons in the diseased brain (Franke et al., 2004; Doná et al., 2009; Engel et al., 2012). Astrocytic and neuronal P2X7R may also contribute to neuronal damage by inducing the release of glutamate and GABA from astrocytes (Wang et al., 2002; Duan et al., 2003; Fu et al., 2013) or from neurons (Wirkner et al., 2005; Marcoli et al., 2008; Cho et al., 2010; Cervetto et al., 2012), unbalancing excitability (Tian et al., 2005) and/or causing a direct neurotoxicity (Volonté et al., 2003) involving either the dilation of P2X7R pore (Di Virgilio et al., 1998; Khadra et al., 2013) or the recruitment of pannexin-1 hemichannels (Suadicani et al., 2012). Accordingly neuronal P2X7R are required for neurotoxicity in HD (Díaz-Hernández et al., 2009), PD (Carmo et al., 2014a) or ischemic conditions (Arbeloa et al., 2012). A direct toxic action of ATP through P2X7R activation has also been shown in oligodendrocytes (Matute et al., 2007), which may be relevant to the contribution of P2X7R to MS (Amadio et al., 2011).

**Figure 2:**
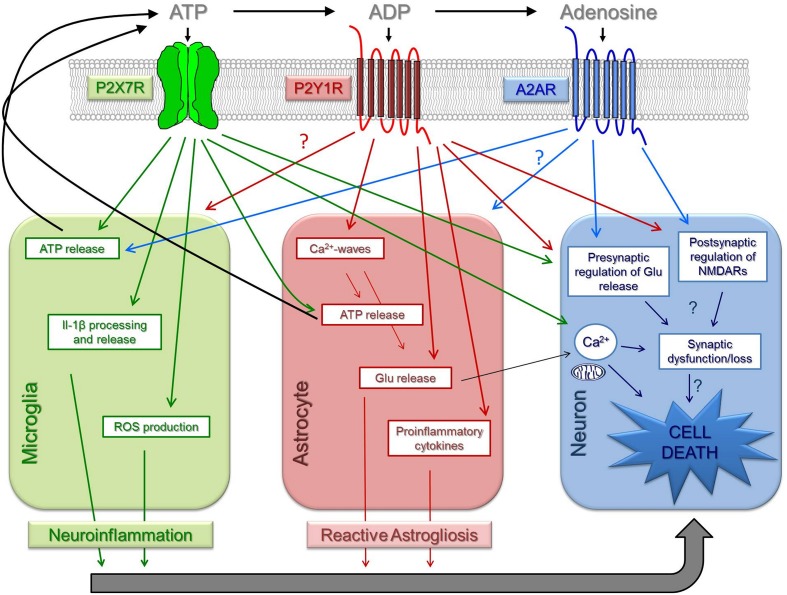
**Schematic diagram of the actions of P2X7R, P2Y1R and A_2A_R in brain pathologies**. Extracellular ATP, both directly through the activation of P2X7R and indirectly through the activation of P2Y1R and A_2A_R upon its extracellular catabolism into ADP and adenosine, seems to be a key signal in brain pathologies, being endowed with the unique capacity to promote and integrate neuroinflammation, reactive astrogliosis, synaptic dysfunction/loss, and increased susceptibility of neurons to damage. Here, it is summarized the different mechanisms reported for each receptor that are or may be contributing to neurodegeneration. The knowledge of the precise mechanisms and the challenging characterization of the temporal and spatial hierarchy of these different actions, perhaps as a common neurodegenerative pathway to different brain disorders, will most likely unravel an opportunity for multi-drug target therapeutics.

In summary, the observed gain of function of P2X7R in pathological conditions, suggests that P2X7R may essentially act as a danger sensor shared by different brain disorders, contributing to the progression of brain diseases through a combined neurotoxic overactivation of microglia, also involving astrocytic-mediated or direct neurotoxic actions (Figures 1, 2).

### P2Y1 receptor

P2Y1R is a metabotropic receptor preferentially activated by ADP, which pharmacological or genetic blockade affords neuroprotection in ischemic conditions (Sun et al., 2008; Kuboyama et al., 2011; Chin et al., 2013; Carmo et al., 2014b) or trauma (Choo et al., 2013). P2Y1R have a widespread cellular distribution and modulate neurons (Bowser and Khakh, 2004; Guzman et al., 2010), astrocytes (Fam et al., 2003; Fumagalli et al., 2003; Zheng et al., 2013) and microglia (Boucsein et al., 2003; Ballerini et al., 2005; Bianco et al., 2005). However, the pathological role of P2Y1R has been predominantly associated to reactive astrocytes since P2Y1R play a key role in entraining the propagation of calcium waves throughout the astrocyte network (Fam et al., 2003; Neary et al., 2003; Bowser and Khakh, 2007) and promote astrocytic hyperactivity and astrogliosis upon mechanical injury (Franke et al., 2001), ischemic conditions (Sun et al., 2008) or AD (Delekate et al., 2014), which is known to interfere with neuronal repair and regeneration (McKeon et al., 1999; Tian et al., 2006). The neuroprotection resulting from P2Y1R blockade might also involve the ability of P2Y1R to control GABA uptake (Jacob et al., 2014) and glutamate release (Domercq et al., 2006) impacting on synaptic function (Jourdain et al., 2007; Santello et al., 2011), and to regulate inflammatory/trophic factors expression in astrocytes (Kuboyama et al., 2011). However, in line with the existence of multiple populations of P2Y1R with different functions in astrocytes operating different transducing pathways (Fam et al., 2003; Sun et al., 2008; Kuboyama et al., 2011; Zheng et al., 2013), the blockade or the stimulation of P2Y1R in astrocytes can cause paradoxical effects; thus, the exogenous overactivation of P2Y1R can prevent astrocytic damage (Shinozaki et al., 2006) and protect against neuronal damage induced by oxidative stress through IL-6 release (Fujita et al., 2009). This apparently paradoxical effect might also result from the up-regulation of P2Y1R in pathological conditions, such as epilepsy (Fernandes et al., 2009; Padrão et al., 2011), mechanical injury (Franke et al., 2004), ischemia (Kuboyama et al., 2011) or AD (Moore et al., 2000), which might trigger a time-dependent gain of noxious function of P2Y1R under non-acute pathological conditions.

Neuronal P2Y1R may also directly affect brain function and damage (Carmo et al., 2014b). P2Y1R are located in central synapses, where they control glutamate release (Mendonza-Fernández et al., 2000; Rodrigues et al., 2005) and NMDA receptors (Luthardt et al., 2003). P2Y1R also control calcium and potassium conductances (Gerevich et al., 2004; Filippov et al., 2006; Coppi et al., 2012) and inhibitory transmission (Bowser and Khakh, 2004; Kawamura et al., 2004), but it is unclear how these different effects impact on the functioning and viability of neuronal networks; in fact, brain insults trigger an up-regulation of neuronal P2Y1R (Moore et al., 2000) coupled to a noxious gain of function, as heralded by the selective ability of P2Y1R to inhibit cortical LTD only in hypoxic conditions (Guzman et al., 2010) and to normalize neurotransmission upon anoxic depolarization (Traini et al., 2011). Finally, microglia P2Y1R are also expected to be involved in the neuroprotection associated with P2Y1R blockade since P2Y1R modulate neuroinflammatory responses (Ballerini et al., 2005). Thus, the role of P2Y1R in neurodegeneration is likely to involve a trans-cellular network, as illustrated by the evidence that activated microglia is capable to modulate synaptic function through ATP release, which in turn stimulates astrocytic P2Y1R controlling glutamatergic gliotransmission that feeds-back to impact on synaptic activity (Pascual et al., 2012) (Figures 1, 2).

In summary, it seems that, in addition to P2X7R, P2Y1R also contribute to brain dysfunction and damage, further arguing for the role of extracellular ATP as a danger signal in brain pathology. This is further heralded by the neurotoxicity of exogenously added ATP (Ryu et al., 2002; Amadio et al., 2005; Resta et al., 2005) and by the neuroprotection afforded by non-selective P2R antagonists (Krügel et al., 2001; Lämmer et al., 2006), supporting that P2R might be valuable targets for neuroprotection (Volonté et al., 2003; Franke et al., 2006).

### A_2A_ receptor

Apart from a direct effect of ATP acting through P2X7R and P2Y1R, ATP may also impact on brain dysfunction upon its extracellular catabolism by ecto-nucleotidases (Cunha, 2001; Zimmermann et al., 2012) into adenosine, followed by activation of adenosine receptors (Cunha, 2005; Chen et al., 2007, 2013; Gomes et al., 2011). In fact, there is robust evidence showing that the pharmacological or genetic deletion of adenosine A_2A_ receptors (A_2A_R) diminishes neurodegeneration and brain dysfunction in animal models of aging (Prediger et al., 2005), PD (Schwarzschild et al., 2006), AD (Canas et al., 2009; Laurent et al., 2014), epilepsy (El Yacoubi et al., 2008, 2009; Cognato et al., 2010), Machado-Joseph's disease (Gonçalves et al., 2013), chronic stress (Batalha et al., 2013) or ADHD (Pires et al., 2009; Pandolfo et al., 2013). This remarkably agrees with the impact of the regular consumption of the non-selective A_2A_R antagonist, caffeine, on age and AD-related memory impairment (Cunha and Agostinho, 2010), PD (Ascherio et al., 2003), and major depression (Lucas et al., 2011). The observation that A_2A_R are mostly located in synapses (Rebola et al., 2005a), A_2A_R selectively control NMDA receptor (Rebola et al., 2008) and synaptic plasticity phenomena (d'Alcantara et al., 2001; Costenla et al., 2011) and the deletion of neuronal A_2A_R is sufficient to afford neuroprotection (Kachroo et al., 2005; Shen et al., 2008; Wei et al., 2014), prompts the hypothesis that the control of synaptotoxicity is at the core of A_2A_R neuroprotection (Cunha and Agostinho, 2010). However, the possible role of A_2A_R in astrocytes (Matos et al., 2012a, 2015; Orr et al., 2015) and in microglia (Orr et al., 2009; Rebola et al., 2011; Gomes et al., 2013) still remains to be determined, especially since A_2A_R undergo a marked up-regulation in neurodegenerative and neuropsychiatric disorders in glial cells (Yu et al., 2008; Matos et al., 2012b) but mainly in synapses (Rebola et al., 2005b; Cunha et al., 2006; Duarte et al., 2012), which is associated with a shift of function of A_2A_R (reviewed in Cunha et al., 2008; Rial et al., 2014) (Figures 1, 2).

Notably, it has been established that the adenosine activating A_2A_R is derived from the activity of ecto-5′-nucleotidase (Cunha et al., 1996b; Rebola et al., 2008; Augusto et al., 2013), the final step in the ATP catabolism into adenosine. Furthermore, unpublished work from our group has documented that the blockade of ecto-5′-nucleotidase or of A_2A_R affords comparable neuroprotection, further heralding the concept that A_2A_R activation is part of the signaling operated by extracellular ATP as a danger signal.

## P2X7R-P2Y1R-A_2A_R: an hazardous orchestra

The sustained increase of extracellular ATP levels upon brain dysfunction/damage together with the compelling evidence that the pharmacological blockade or genetic deletion of P2X7R or P2Y1R or A_2A_R prevents or attenuates neuronal injury or the onset/evolution of brain diseases, supports a role for ATP both as a warning and harmful signal in the brain. It will now be important to understand the time-dependent involvement of these three purinoceptors and their inter-play. In fact, the activation of A_2A_R or P2X7R may constitute an auto-stimulatory loop (Verderio and Matteoli, 2001; Cunha et al., 2012) since they can trigger ATP release from astrocytes, neurons or microglia (George et al., 2015), either directly through the P2X7R pore (Duan and Neary, 2006), through interaction with pannexin channels (Locovei et al., 2007; Iglesias et al., 2008; Bennett et al., 2012), or by exocytotic release (Gutiérrez-Martín et al., 2011). Furthermore, P2X7R synergistically regulate P2Y1R activation (Locovei et al., 2006), particularly in pathological conditions (Traini et al., 2011; Vessey et al., 2011; Choo et al., 2013). Finally, emerging evidence indicates a synergic interplay between ATP and its metabolite adenosine (Gerwins and Fredholm, 1992; Neary et al., 1998; Chevrier et al., 2006; Färber et al., 2008; Koizumi et al., 2013; George et al., 2015), namely between A_2A_R and P2X7R (Chen et al., 2004; Pellegatti et al., 2011) and P2Y1R (Stafford et al., 2007; Doengi et al., 2008; Suzuki et al., 2011), which highlights the possible key role of ecto-nucleotides in regulating the integration of purinergic responses. Thus, the action of individual purinergic receptors may be part of a time-dependent orchestrated response triggered by the increase of extracellular ATP levels in brain pathology (Figure 2). The understanding of the hierarchy and integration/redundancy of their actions will be paramount to develop multi-target therapeutics to exploit this role of ATP as a danger signal in the brain.

### Conflict of interest statement

The authors declare that the research was conducted in the absence of any commercial or financial relationships that could be construed as a potential conflict of interest.
